# Ayu-Characterization of healthy aging from neuroimaging data with deep learning and rsfMRI

**DOI:** 10.3389/fncom.2022.940922

**Published:** 2022-09-12

**Authors:** Kushal Borkar, Anusha Chaturvedi, P. K. Vinod, Raju Surampudi Bapi

**Affiliations:** iHub-Data, International Institute of Information Technology, Hyderabad, India

**Keywords:** rs-fMRI, attention, static functional connectivity matrix, age estimation, interpretability, classification, regression

## Abstract

Estimating brain age and establishing functional biomarkers that are prescient of cognitive declines resulting from aging and different neurological diseases are still open research problems. Functional measures such as functional connectivity are gaining interest as potentially more subtle markers of neurodegeneration. However, brain functions are also affected by “normal” brain aging. More information is needed on how functional connectivity relates to aging, particularly in the absence of neurodegenerative disorders. Resting-state fMRI enables us to investigate functional brain networks and can potentially help us understand the processes of development as well as aging in terms of how functional connectivity (FC) matures during the early years and declines during the late years. We propose models for estimation of the chronological age of a healthy person from the resting state brain activation (rsfMRI). In this work, we utilized a dataset (*N* = 638, age-range 20–88) comprising rsfMRI images from the Cambridge Centre for Aging and Neuroscience (Cam-CAN) repository of a healthy population. We propose an age prediction pipeline Ayu which consists of data preprocessing, feature selection, and an attention-based model for deep learning architecture for brain age assessment. We extracted features from the static functional connectivity (sFC) to predict the subject's age and classified them into different age groups (young, middle, middle, and old ages). To the best of our knowledge, a classification accuracy of 72.619 % and a mean absolute error of 6.797, and an *r*^2^ of 0.754 reported by our Ayu pipeline establish competitive benchmark results as compared to the state-of-the-art-approach. Furthermore, it is vital to identify how different functional regions of the brain are correlated. We also analyzed how functional regions contribute differently across ages by applying attention-based networks and integrated gradients. We obtained well-known resting-state networks using the attention model, which maps to within the default mode network, visual network, ventral attention network, limbic network, frontoparietal network, and somatosensory network connected to aging. Our analysis of fMRI data in healthy elderly Age groups revealed that dynamic FC tends to slow down and becomes less complex and more random with increasing age.

## 1. Introduction

The process of aging is gradual and multi-factorial leading to the loss of biological and physical function. It can be influenced by several factors including environmental and biochemical mechanisms. The human brain structure and function are continuously evolving with aging. Estimating brain age may help to study the deviation from the trajectory of healthy aging. Neuroimaging data can help in brain age estimation and extracting relevant biomarkers of healthy aging and brain disorders.

Functional brain connectivity (FC) allows us to investigate how functionally distinct regions of the brain interact with one another. Resting-state functional magnetic resonance imaging (rs-fMRI) has become one of the most important modalities to examine the human brain's functional connectivity (Greicius et al., 2003; Fox et al., 2009; Biswal et al., 2010; Smith et al., 2011). rs-fMRI analysis has helped to understand the difference in functional connectivity between healthy and disease conditions (Dosenbach et al., 2010; Erus et al., 2015). Such data also captures the functional changes of the aging process. The information contained in such data is complex, requiring the adoption of approaches such as machine learning and deep learning to develop predictive models of brain disorders.

Past studies have utilized various neuroimaging modalities such as EEG (Al Zoubi et al., 2018), diffusion tensor imaging (Mwangi et al., 2013), unprocessed T1-weighted (T1w) structural MRI (Cole et al., 2015, 2017; Jiang et al., 2020), and rs-fMRI (Li et al., 2018; Monti et al., 2020) for brain age estimation. The models proposed in these studies for estimation of brain age are mainly statistical models built on a healthy aging population. These models vary in complexity as well as in the class of neuroimaging data employed (Cole et al., 2015). In recent years, sophisticated machine learning and deep learning methods have been used for brain age estimation and brain age classification (Cole et al., 2017; Lancaster et al., 2018). The study of Cole et al. (2017) and Jiang et al. (2020) utilize structural magnetic resonance imaging (MRI) for brain age prediction results using deep learning approaches such as CNN-based studies. The CNN-based results showed age prediction is fundamentally consistent with the changing pattern of the gray matter volume with age.

Brain age prediction has been demonstrated by applying CNNs in brain networks in neurodevelopment (Kawahara et al., 2017; Li et al., 2018). There is a need to work on adequately diverse datasets so that every possible age group is considered while modeling the aging phenomenon. However, the past studies have generally centered around resting-state information for a particular age group. The contrast between different age groups in resting-state information is significant in the investigation of aging as age-related cognitive and neural differences (Davis et al., 2014). Furthermore, aging studies concentrate on younger and older adults, leaving out the moderate age group which represents a significant amount of the adult lifespan.

Significantly, the static functional connectivity network (sFC) of the brain changes with aging and there might be individual variations in these patterns from subject to subject. However, we highlight here the broad consensus patterns of change that are emphasized in the literature. Differential impact of aging on specific brain networks has been investigated previously (Ferreira and Busatto, 2013; Dennis and Thompson, 2014; Sala-Llonch et al., 2015). Most of the studies have targeted the default-mode network (DMN), showing lower functional connectivity between its different regions of interest (ROIs) with aging (Damoiseaux et al., 2008), other studies also focus on age effects in other brain ROIs networks, e.g., salience and sensorimotor networks (Meier et al., 2012; He et al., 2014; Geerligs et al., 2015; La Corte et al., 2016).

Critically, previous studies on functional connectivity in normal aging were led with relatively small samples or included wide age ranges instead of middle age and older age groups. In particular, we need to understand the aging influences relations between functional network properties and consider all the age groups for the generalizability of the findings. Restricting analyses to an individual resting-state network, like the DMN, might be inadequate in acquiring a more comprehensive understanding of the functional connectivity of the aging brain. Finally, previous studies characterized networks dependent on anatomical parcellations that do not necessarily conform to the true functional architecture of the human brain (Wang et al., 2010). The purpose of this study is to identify the functional connections that distinguish older adult brains from younger adult brains including the middle-aged group.

In this study, we propose an age prediction pipeline *Ayu - “age” and “longevity” in Sanskrit* which consists of data preprocessing, feature selection, and an attention-based residual network for deep learning architecture for brain age assessment from rs-fMRI. Based on the age prediction pipeline Ayu implemented on aging rs-fMRI data, we present our results to predict the subject age and classify them into different age groups (young, middle, middle-old, and old ages). Our model performs well in the hold-out test set from the same population. We demonstrate how the correlation between different functional regions changes with age by extracting features from the static functional connectivity (sFC) by applying attention-based networks and activation maps. The contributions of the brain regions that underwent significant age-related changes for the classifier to discriminate between the four age groups can be accurately assessed and identified by observing the integrated gradient maps for each class obtained from the network. To understand the changes in resting static functional networks, we analyze large-scale networks in the entire brain and allow for both decreases and increases in connectivity obtained from the integrated gradient results. We hypothesized that in the middle-aged group from the general population, networks showing an increase in functional connectivity would most likely be those previously implicated in aging. We aim to distinguish the relevant FC with significant developmental trends that could facilitate our understanding of functional brain development.

## 2. Materials and methodology

In this section, we discuss our framework *Ayu*, which includes the various steps: (i) data curation and preparation, (ii) data pre-processing, and (iii) experimental setup used for brain age estimation using the resting-state fMRI modality. Moreover, we applied an attention-based mechanism to interpret the estimated brain age.

### 2.1. Data curation and preparation

We worked with the data of healthy participants obtained from the Cambridge Centre for Aging and Neuroscience (Cam-CAN) repository for brain age assessment from rs-fMRI (Taylor et al., 2017).

Participants were co-registered to that participant's T1-weighted image *via* a rigid-body (6-df) linear transformation, and normalization parameters from the DARTEL procedure were applied in the normalization stages of the other streams. This establishes the six motion parameters for motion correction and removes residual motion artifacts using wavelet de-spiking (Patel et al., 2014), regression of WM/CSF signals, and higher-order expansions of the movement parameters (Geerligs et al., 2015). This procedure ensures a voxel-to-voxel correspondence for all metrics derived from the BOLD time series (which is used in rsfMRI).

rsfMRI Data used in CamCAN were unwarped (using field-map images) to compensate for the magnetic field inhomogeneities, realigned for motion, and slice-time corrected. After the EPI data were co-registered to the T1 image, the normalization parameters from the VBM stream were then applied to warp functional images into MNI space (Taylor et al., 2017).

The rs-fMRI scans were obtained while participants were resting with their eyes closed in a 3T Siemens TIM Trio scanner and a total of 261 whole-brain volumes were acquired (please see Taylor et al., 2017). For more details on the acquisition parameters) with a 32-channel head coil with the acquisition parameters: TR/TE = 1,970/30 ms, 32 pivotal cuts, flip point =78°; FOV =192 mm × 192 mm; voxel-size = 3 mm × 3 mm × 4.44 mm, acquisition time = 8 min 40 s, number of volumes: 261 with their eyes closed (Taylor et al., 2017).

### 2.2. Data processing

The rsfMRI data was pre-processed using an optimized procedure, including min-max scaling and the regression after the processing done by Taylor et al. (2017). rs-fMRI images were collected from 638 healthy participants with the participants' age ranging from 20 to 88 years (average of 54.94 ± 18.02; 315 men and 323 women). They were divided into 4 age groups: young (172 subjects from 20 to 40 yrs), adult (152 subjects from 41 to 55 yrs), middle-old (154 subjects from 56 to 69 yrs), and old (160 subjects from 70 to 88 yrs), thus giving us a near-uniform distribution of the number of participants across different age groups. We exclude subject below 20 years and above 88 years.

#### 2.2.1. Functional connectivity

To calculate an ROI-based whole-brain functional connectivity, we used two brain atlases for parcellation of the subject's brain scan, (i) Schaefer Atlas which consists of a 100-region of 17-network Schaefer parcellation, and (ii) BASC Multiscale Atlas which consists of 64-region BASC Multiscale parcellation, to obtain functionally meaningful averaged BOLD signals for each measurement. We used nilearn to extract the static functional connectivity (Pedregosa et al., 2011) from the rsfMRI scans. For static Functional Connectivity networks (sFC), pairwise Pearson correlation coefficient was calculated between the different brain regions for the time series signals obtained from each brain scan. To obtain a symmetrical correlation matrix, we calculated full connectivity matrices leading to 100 × 100 for Schaefer Atlas (Schaefer et al., 2018) and 64 × 64 for BASC Multiscale Atlas (Bellec et al., 2010) connectivity features and the average signal for each region was used to obtain functional connectivity (FC) matrix.

### 2.3. Experimental setup

We created training and test sets of participants for model training and evaluation. We performed stratified split-based brain scans with an 80:20 ratio for training and testing sets, respectively, ensuring the uniform distribution of age, gender, and brain scans in the training and test sets.

We initialized our model with Kaiming initialization (He et al., 2015), which provided us with stable gradients throughout the network. This helped us initialize weight with a normal distribution with a mean of 0 and variance std and the ideal distribution of weight after ReLU should have slightly incremented mean layer by layer and variance close to 1. It is a common practice to use Kaiming initialization as it shows better stability than random initialization.

### 2.4. Ayu-Pipeline

In this section, we propose Ayu Pipeline employed in the classification and the brain age estimation task. We first standardize the training data and the testing data by transforming the mean and SD as 0 and 1, respectively. This helped us to transform all the sFC matrices into the same scale.

For machine learning algorithms, the matrices obtained from two atlases were converted to vectors by linearizing the lower triangular matrix without the diagonal. We used two machine learning algorithms to classify the subject in one of the 4 age groups namely young, middle, middle-old, and old: Support Vector Classifier and Linear Discriminant Analysis from python sci-kit learn toolbox (Pedregosa et al., 2011). Both these algorithms are parameter-free algorithms.

We used the correlation-based static Functional Connectivity (sFC) as an input to the three deep learning algorithms namely AlexNet, VGGNet5, and ResNet (Krizhevsky et al., 2012; Simonyan and Zisserman, 2014; He et al., 2016). We used only 5 layers hence the name, VGG5 and ResNet. For VGG, we use the general CNN architecture similar to the VGG classification architecture (Simonyan and Zisserman, 2014) which consists of interleaved convolutional blocks followed by max-pooling layers. For convolutional layers, we considered a convolutional kernel size of 3 × 3, a batch size of 16, rectified linear unit (ReLU) as the activation functions. We flatten the output from the last convolutional layer and feed it into a fully-connected (FC) layer with softmax as the activation. For ResNet, the deep learning model contains one convolution layer, followed by 4 residual blocks, 1 fully connected layer, and an output layer. The same configuration was followed by convolutional layers with a convolutional kernel size of 3 × 3, a batch size of 16, rectified linear unit (ReLU) as the activation function.

**Figure 1:**
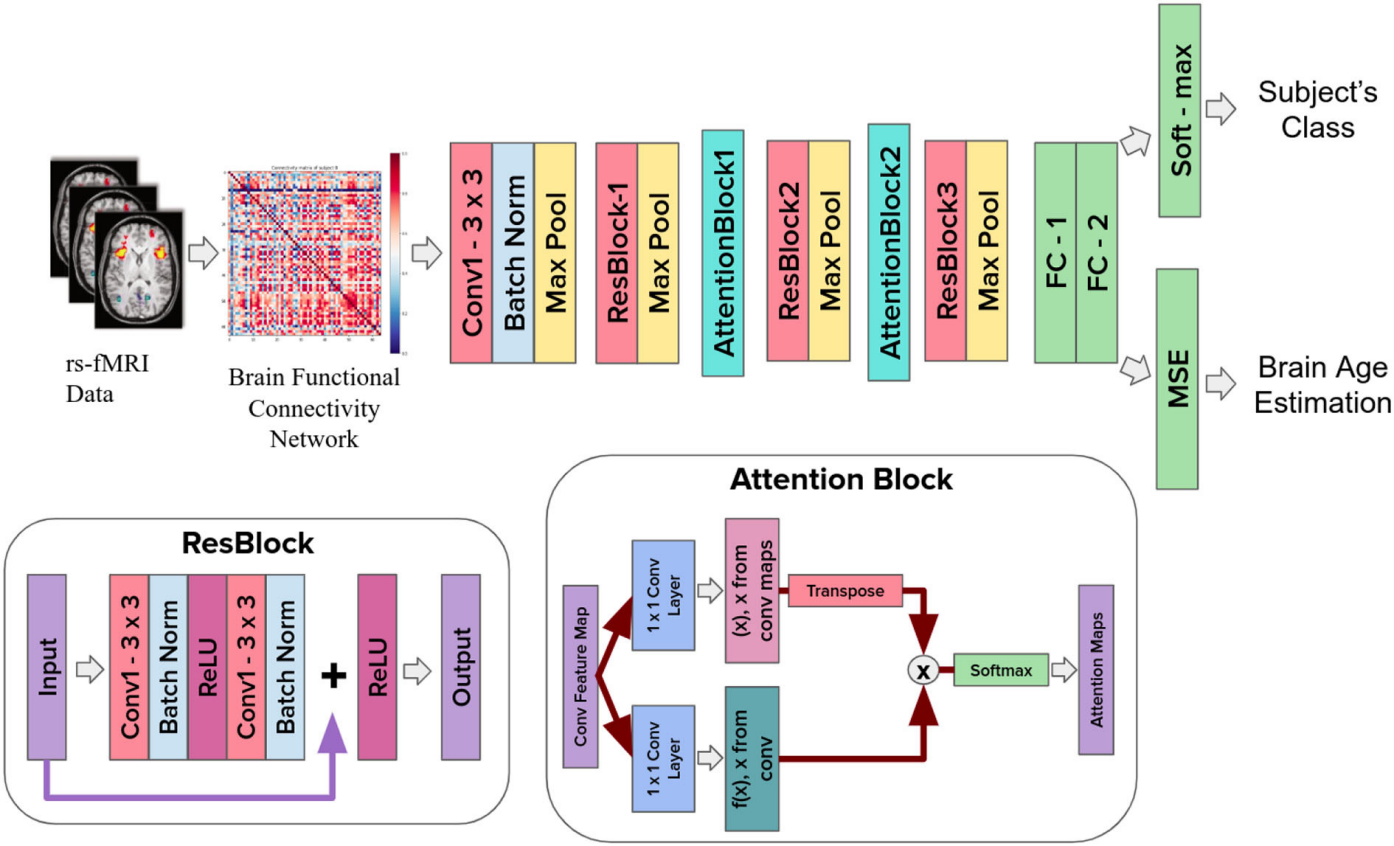
Ayu pipeline which includes ResNet with attention.

For Regression, we used three machine learning algorithms to estimate the brain age: Regularized linear regression (ElasticNet), Support Vector Regression, and Bayes Ridge Regression. We used python's sci-kit learn toolbox (Pedregosa et al., 2011) to get the results. The same algorithms were used for deep learning architecture but we implemented linear operation for our activation function instead of a softmax in the classification task (Figure 1).

#### 2.4.1. Attention mechanism

The attention model was inspired by Jetley et al. (2018) and Zhang et al. (2019), where authors introduced an attention-based mechanism for the classification of the computer-vision based task.

Here, the approach we followed is based on covariance between the predicted region of interest with respect to every region in the functional connectivity matrix, (where each region is a random variable). The attended target region is a weighted summation of all the correlation values of the functional connectivity matrix.

The matrix features $f_{i}^{l}$ extracted at a given layer, l ∈ {1, 2, …, *L*} represents the pixel-wise feature vectors. The global feature vector *g* extracted before the final layer of the classifier and regressor encodes the global, discriminative, relevant ROIs from the sFC. The compatibility score $c_{i}^{l}$ is defined as follows:


(1) $$\begin{array}{r} c_{i}^{l}=\langle \lambda^{l},f_{i}^{l}+g\rangle \end{array}$$

Here, the weight vector λ can be interpreted as learning the set of features to get the relevant features in the sFC. λ shows how the network adjusts its focus according to the context. The attention takes a sequence of vectors as an input for each FC matrix and returns an “attention" vector for the relevant ROIs in the FC matrix.

We apply the proposed attention mechanism to layers 3 and 4 prior to pooling, for both the VGG and the ResNet architectures. Once the attention map is obtained, the weighted average over the spatial axes is computed for each channel in the feature map. The proposed attention mechanism is incorporated in the **Ayu** Pipeline to better exploit local information present in the input sFC matrix which helps in getting the distinct features. Let the output of the attention blocks 1 and 2 be, *M*(*x*) and *N*(*x*), respectively. *M*(*x*) and *N*(*x*) can be represented as:


(2) $$\begin{array}{r} \begin{array}{c} M\left(x\right)=W_{m}\left(x\right),\;N\left(x\right)=W_{n}\left(x\right) \end{array} \end{array}$$

where $W_{m},W_{n}\in R^{\left(c\times ratio\times c\right)}$ are the attention block parameters, *c* is the number of channels for input from attention blocks 1 and 2 and *ratio* is a proportional coefficient, which can be assigned as 1/2, 1/4 progressively for subsequent attention layers. We performed a transpose operation on *M*(*x*) and *N*(*x*) to meet the requirement of the matrix multiplication operation. The attention weight vector λ is obtained through the Softmax layer as shown in Equation (3):


(3) $$\begin{array}{r} \begin{array}{c} \lambda^{l}=\frac{\exp\left(f_{i}^{l}+g\right)}{\sum_{i=0}^{C}\exp\left(f_{i}^{l}+g\right)}\\ where,f_{i}^{l}=M\left(x_{i}\right)N{\left(x_{j}\right)}^{T} \end{array} \end{array}$$

## 3. Results

Our study is based on the hypothesis that brain age shows subtle changes within a smaller age range as compared to a larger age range. Taking this hypothesis into consideration, we perform a 4-class classification which is further followed by a brain age prediction task.

### 3.1. Experimental result

#### 3.1.1. Classification

Table 1 shows the brain age classification results for Schaefer Atlas and BASC Multiscale atlas using both the machine learning and the deep learning models. These outcomes showed that the prediction models based on the whole-brain FC measures outperformed those built upon the coarse-grained FC measures between brain regions or intrinsic connectivity networks (ICNs) (Li et al., 2018). This shows that the FC measures of the entire brain are more informative for the brain age prediction task. Among the proposed deep learning models, the outcomes were improved after applying the attention mechanism. The attention network interprets the important regions in the connectivity matrix. This demonstrates that the hierarchical features learned by the deep learning model better characterize brain developmental information. The best performing model ResNet successfully discriminated 4 classes namely young, adult, middle-old, and old giving an accuracy of 72.619%.

**Table 1:** Classification results based on Schaefer and BASC multiscale Atlas among 4 different classes.

	**Accuracy using**	**Accuracy using**
**Algorithm used**	**Scheafer**	**BASC multi-scale**
	**Atlas**	**Atlas**
Support vector classifier	42.578%	40.578%
Linear discriminant analysis	43.359%	41.359%
AlexNet	50.612%	49.612%
VGGNet5	63.750%	59.750%
ResNet5	66.806%	63.806%
VGGNet5 with attention	68.571%	65.571%
**ResNet5 with attention**	**72.619%**	**69.619%**

The bold values represent the results obtained using the Ayu pipeline.

To check whether we are dealing with the problem of over-fitting, we also present our results with stratified 5-fold cross-validation (each set contains approximately the same percentage of samples of each target class as the complete set) on the same dataset in Table 2.

**Table 2:** Classification results with stratified 5-fold cross-validation using ResNet5 with attention based on Schaefer and BASC multiscale Atlas among 4 different classes.

**ResNet5 with**	**Accuracy using**	**Accuracy using**
**Attention**	**Scheafer**	**BASC multi-scale**
	**Atlas**	**Atlas**
Fold 1	73.001%	69.721%
Fold 2	72.291%	69.551%
Fold 3	72.599%	69.599%
Fold 4	72.621%	69.629%
Fold 5	72.649%	69.612%
**Mean result**	**72.632%**	**69.622%**
**Results on test data**	**72.619%**	**69.619%**

The bold values represent the results obtained using the Ayu pipeline.

We also compared the quantitative evaluation results with the state of the methods in Table 3.

**Table 3:** Comparison of different state-of-the-art approaches with Ayu for the brain age classification task.

**Methods**	**Features used**	**Accuracy**
Meier et al. (2012)	Seed based regions	43.012%
Tian et al. (2016)	Linked independent components	53.910%
Li et al. (2018)	Whole brain FC	69.086%
Monti et al. (2020)	Linear latent variable model	57.482%
**Our result**	Whole brain FC using scheafer atlas	**72.619%**

The bold values represent the results obtained using the Ayu pipeline.

#### 3.1.2. Regression

A total of 638 participants were considered with the minimum age being 20 years. The chronological age was used as a training criterion for evaluating our results. Table 4 shows the *R*^2^, MAE, and RMSE values between the chronological age and the predicted age in the testing dataset. The best performing model ResNet with Attention effectively provided higher *r*^2^, MAE, and RMSE of 0.754, 6.797, and 8.002, respectively. Table 5 shows our results with stratified 5-fold cross-validation using ResNet with Attention architecture.

**Table 4:** *R*^2^, *MAE*, and *RMSE* values using different state-of-the-art methods.

**Algorithm used**	**Scheafer atlas**			**BASC multiscale atlas**		
	***r* ^2^**	***MAE***	***RMSE***	***r* ^2^**	***MAE***	***RMSE***
Elastic regression	0.613	11.923	13.856	0.592	13.856	17.589
Support vector regressor	0.615	10.199	11.923	0.617	9.963	11.923
Bayes ridge regression	0.607	9.457	11.706	0.619	9.457	11.706
AlexNet	0.618	9.725	11.342	0.617	10.515	12.375
VGGNet5	0.671	8.439	10.016	0.624	10.018	12.048
ResNet5	0.724	7.869	9.002	0.662	9.272	11.863
VGGNet5 with Attention	0.721	7.310	9.316	0.674	9.048	11.121
**ResNet5 with attention**	**0.754**	**6.797**	**8.002**	**0.712**	**8.272**	**10.986**

The bold values represent the results obtained using the Ayu pipeline.

**Table 5:** *R*^2^, *MAE*, and *RMSE* values with stratified 5-fold cross-validation using ResNet5 with Attention.

**ResNet5 with Attention**	**Scheafer atlas**			**BASC multiscale atlas**		
	***r* ^2^**	***MAE***	***RMSE***	***r* ^2^**	***MAE***	***RMSE***
Fold 1	0.7511	6.7971	7.9841	0.7179	8.2717	10.9717
Fold 2	0.7601	6.7984	7.9792	0.7084	8.2790	11.0002
Fold 3	0.7544	6.7898	8.0580	0.7258	8.2725	10.8800
Fold 4	0.7539	6.8039	7.9644	0.7209	8.2730	10.9272
Fold 5	0.7521	6.7964	8.0190	0.7294	8.2699	10.9269
**Mean result**	**0.7543**	**6.7972**	**8.0008**	**0.7204**	**8.2734**	**10.9412**
**Results on test data**	**0.754**	**6.797**	**8.002**	**0.712**	**8.272**	**10.986**

The bold values represent the results obtained using the Ayu pipeline.

We also compared our model performance with other methods for age prediction in Table 6. Ayu yielded the MAE, RMSE, and *r*^2^ of 6.7, 8.0, and 0.754, respectively, which are better when compared with the deep learning CNN model proposed for age prediction using rs-fMRI (7.9, 10.01, and 0.661, respectively) (Li et al., 2018) and linear latent variable model (12.3, 14.01, and 0.587, respectively) (Monti et al., 2020).

**Table 6:** *R*^2^, *MAE*, and *RMSE* values using Algorithms used for both Schaefer Atlas and BASC Multiscale Atlas.

**Methods**	**Features used**	**Results**		
		***r* ^2^**	***MAE***	***RMSE***
Meier et al. (2012)	Seed based regions	0.551	12.857	13.906
Tian et al. (2016)	Linked independent components	0.564	12.925	14.042
Li et al. (2018)	Whole Brain FC	0.661	7.910	10.016
Monti et al. (2020)	Linear latent variable model	0.587	11.397	13.012
**Our results**	Whole brain FC using scheafer atlas	**0.754**	**6.797**	**8.002**

The bold values represent the results obtained using the Ayu pipeline.

**Figure 2:**
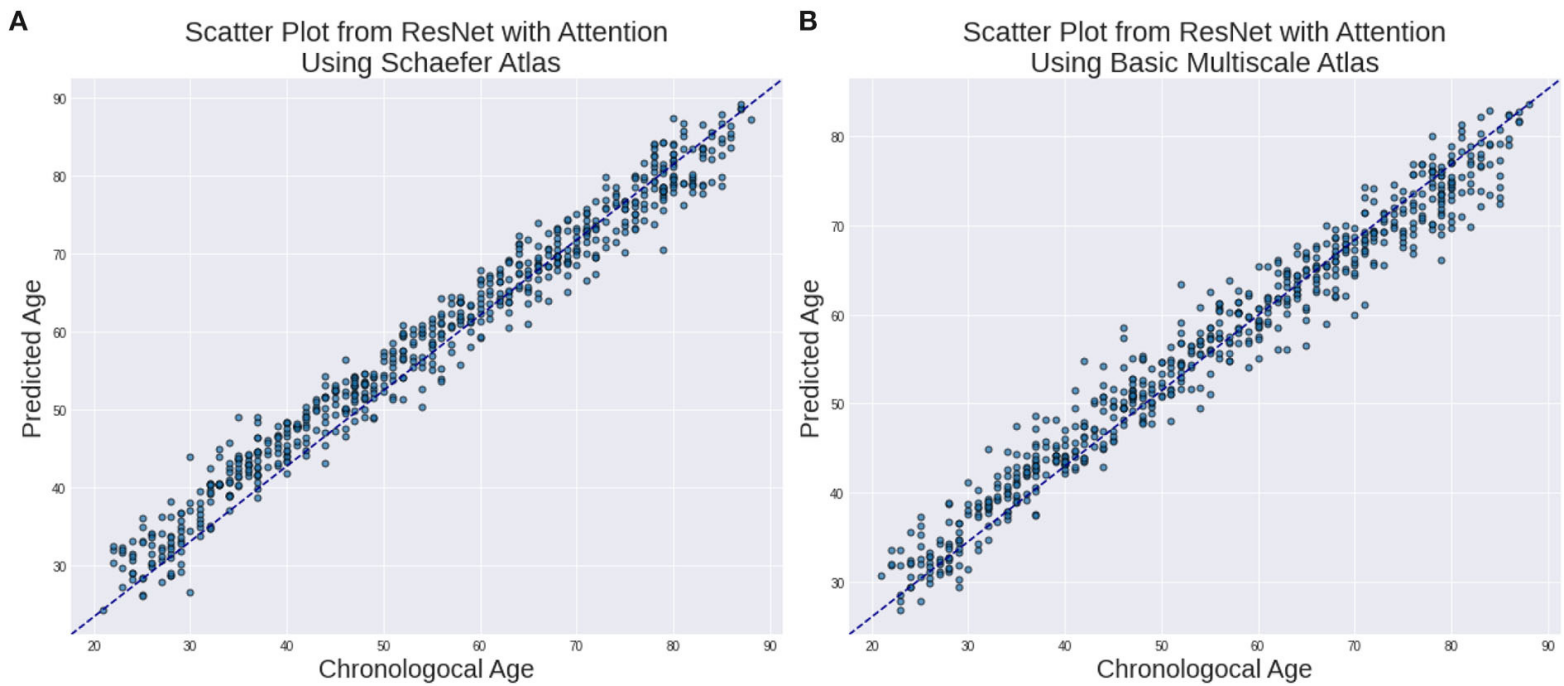
Estimated Age vs. Chronological age using ResNet with Attention for **(A)** Schaefer Atlas and **(B)** BASC Multiscale Atlas lifespan.

In Figure 2, we show the relationship between the estimated age and chronological age for both the atlases.

### 3.2. Interpretability

To identify brain regions that undergo significant age-related changes in functional connectivity, the contribution of each region to the overall ability of the classifier to accurately discriminate between age groups can be assessed by observing the integrated gradient map for each class obtained from the functional connectivity matrices.

#### 3.2.1. Integrated gradient

Integrated Gradient is a technique for attributing a model prediction to its input features. It is a model interpretability technique: one can use it to visualize the relationship between input features and the model predictions (Nain, 2020). It requires no modification in the original network, is easy to implement, and is applicable to a variety of deep models (both sparse and dense). The method works by taking derivatives of the value for the predicted class with respect to the input features (Sundararajan et al., 2017).

For the integrated gradient, we start with the baseline connectivity matrix with all the values as zero and generate a linear interpolation between the baseline and the original connectivity matrix. We calculate gradients to measure the relationship between changes to a feature and changes in the model's predictions. The gradient informs which ROI in the connectivity matrix has the strongest effect on the model's predicted class probabilities. Table 7 lists the features and their relative weights or contributions obtained after implementing the integrated gradient from our method using the Schaefer Atlas.

**Table 7:** Strength^*^ of the relative positively correlated ROI 1 connected with ROI 2 obtained from Integrated Gradient in Schaefer Atlas.

**ROI 1**	**with**	**ROI 2**	**Young**	**Adult**	**Middle-Old**	**Old**
LH Default PFC 1		LH SomMot 2	0.61	0.56	0.57	0.49
LH Default PFC 3		LH Default Par 1	0.39	0.47	0.45	0.40
LH Default Par 1		RH Default Par 2	0.47	0.54	0.57	0.52
LH Default PFC 2		RH Default PFCv 1	0.60	0.57	0.57	0.51
LH Default PFC 1		RH Vis 1	0.38	0.43	0.42	0.41
LH Default PFC 3		LH SomMot 4	0.50	0.51	0.54	0.52
LH SomMot 5		RH SalVentAttn ParOper 1	0.41	0.46	0.47	0.39
LH Default PFC 5		RH SalVentAttn ParOper 1	0.43	0.45	0.48	0.44
LH SalVentAttn PFCl 1		LH Default Par 2	0.33	0.38	0.41	0.37
LH Limbic OFC 1		RH Vis 2	0.52	0.56	0.57	0.49
RH Limbic TempPole 1		RH SalVentAttn Med 1	0.42	0.46	0.45	0.39
LH Limbic TempPole 2		RH Vis 5	0.65	0.56	0.57	0.49
LH Limbic OFC 1		RH Limbic TempPole 1	0.65	0.56	0.57	0.49
LH SomMot 2		LH Default PFC 4	0.54	0.58	0.55	0.50
LH SomMot 4		RH Default PFCv 2	0.57	0.53	0.53	0.49
RH SomMot 3		RH SalVentAttn ParOper 2	0.42	0.45	0.47	0.41
RH SomMot 1		LH Default PFC 4	0.49	0.49	0.46	0.42
LH SomMot 6		RH SomMot 3	0.46	0.45	0.42	0.40
LH Default Par 1		RH Default PFC 2	0.54	0.56	0.53	0.49
LH Default Par 2		RH SomMot 3	0.56	0.57	0.57	0.51
LH Default Par 2		LH Default pCunPCC 2	0.55	0.58	0.55	0.52
RH Vis 3		RH Vis 4	0.65	0.56	0.57	0.49
RH Vis 2		LH SomMot 2	0.65	0.56	0.57	0.49
RH Vis 3		LH Default PFC 2	0.41	0.44	0.48	0.45
RH Vis 4		LH Default PFC 3	0.39	0.42	0.46	0.43
RH Vis 5		RH Default Par 2	0.35	0.38	0.42	0.40
RH Vis 4		LH Default PFC 1	0.36	0.41	0.45	0.43

LH, Left Hemisphere; RH, Right Hemisphere.

* Omitted values with a weight less than 0.2.

In practice, computing a definite integral is not always numerically possible and can be computationally costly, so the following numerical approximation is computed (Sundararajan et al., 2017):


(4) $$\begin{array}{l} IntegratedGradient_{i}^{approx}(x)\approx \left(x_{i}-{x^{\prime }}_{i}\right)\\ \;\times \sum_{k=1}^{m}\frac{\partial F\left(x^{\prime }+\frac{k}{m}\times \left(x_{i}-{x^{\prime }}_{i}\right)\right)}{\partial x_{i}} \end{array}$$

where *i* denotes the individual ROI feature; *x* denotes the input tensor; *x*′ baseline tensor; *k* denotes scaled feature perturbation constant; and *m* denotes the number of steps in the sum approximation of the integral.

**Figure 3:**
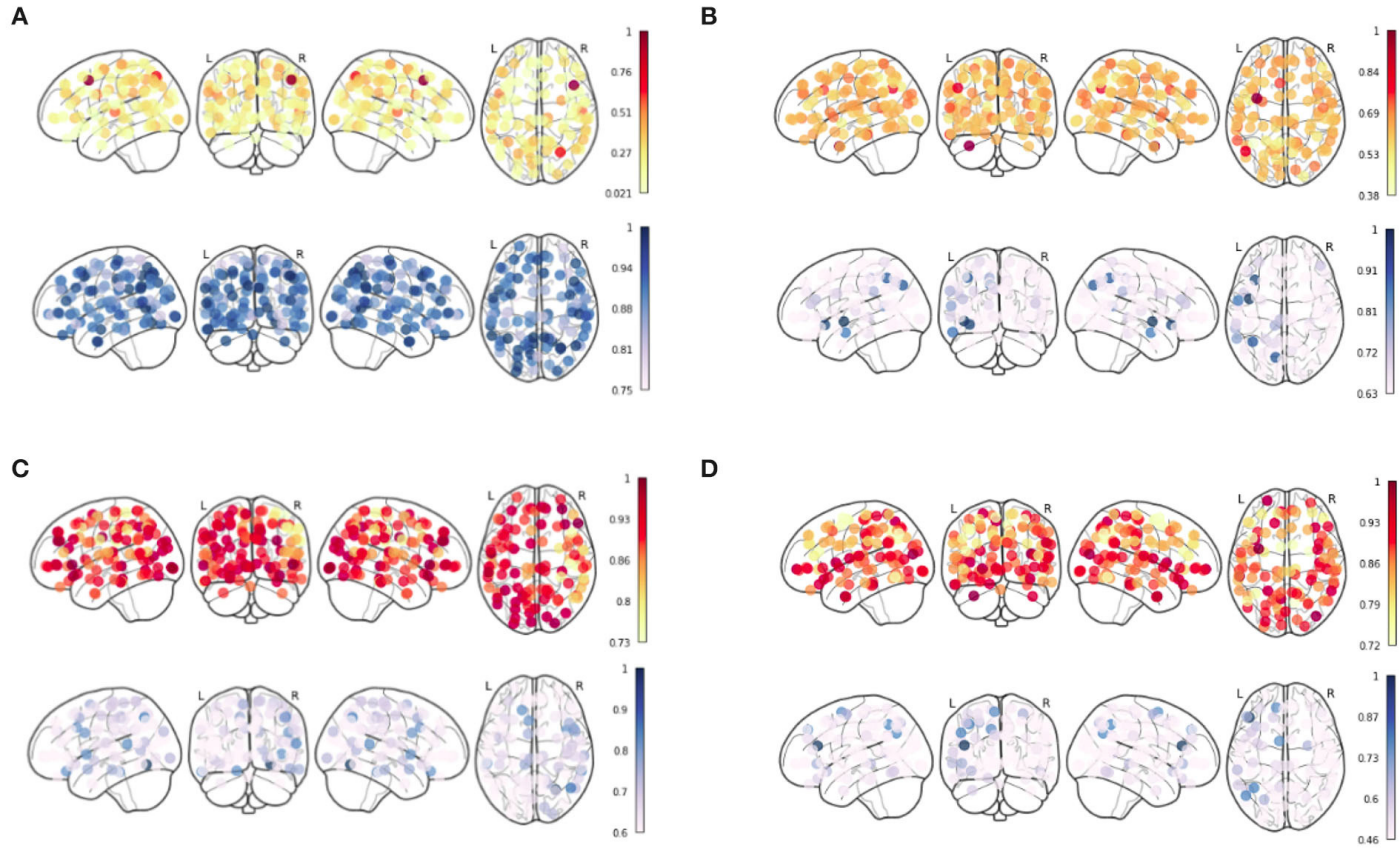
Inferred Node Strength from Ayu Pipeline over the sFC from Schaefer Atlas after applying Integrated Gradient for **(A)** Young, **(B)** Adult, **(C)** Middle-Old Class, and **(D)** Old Class recovered.

To observe the pattern which can characterize the 4 age groups, we generate the Node Strength for their respective age groups using the results which were obtained from the integrated gradient. The results for the four age groups are shown in Figure 3. The red color bar corresponds to the positive inferred node strength whereas the blue color bar corresponds to the negative inferred node strength obtained from the mean functional connectivity matrix from each age group. Our analysis of the inferred node strength shows that the node strength for the positive edges is the highest in the middle-old class and the lowest in the young age group (refer to Figure 3). This further shows that the positive inferred nodes increase as we go from the young to the middle-old age group.

To detect the change within brain networks, we analyzed the node weights obtained by the individual networks of the brain region on the entire cohort from the integrated gradient. This helps us understand the change across the node strength of the individual's functional region in the static functional connectivity with aging. Table 8 shows the node strength of individual functional brain regions along with the corresponding ROIs using the Schaefer Atlas.

**Table 8:** Contribution of all the features and their corresponding Brain Region Obtained from Integrated Gradient in Schaefer Atlas.

**Brain**	**ROI in**	**Young**	**Adult**	**Middle-Old**	**Old**
**region**	**Scheafer Atlas**			
	LH Default PFC 1	0.632	0.551	0.556	0.512
	LH Default PFC 2	0.648	0.602	0.591	0.523
Pre-Frontal	LH Default PFC 3	0.524	0.541	0.517	0.489
Cortex Network	LH Default PFC 4	0.557	0.523	0.462	0.408
	LH Default PFC 5	0.462	0.481	0.436	0.369
	RH Default PFC 1	0.347	0.312	0.274	0.241
	RH Default PFC 2	0.326	0.302	0.281	0.267
	LH SomMot 2	0.353	0.391	0.406	0.381
	LH SomMot 4	0.332	0.361	0.326	0.291
Somatosensory	LH SomMot 5	0.375	0.364	0.363	0.345
Network	LH SomMot 6	0.392	0.381	0.358	0.331
	RH SomMot 1	0.292	0.326	0.319	0.262
	RH SomMot 3	0.306	0.341	0.325	0.297
	LH SalVentAttn PFCl 1	0.326	0.384	0.363	0.311
	RH SalVentAttn Med 1	0.342	0.391	0.387	0.361
Ventral Attention	RH SalVentAttn ParOper 1	0.352	0.387	0.373	0.336
Network	RH SalVentAttn ParOper 2	0.332	0.380	0.388	0.378
	RH SalVentAttn TempOccPar 1	0.285	0.324	0.340	0.336
	RH SalVentAttn FrOperIns 1	0.263	0.304	0.316	0.306
	LH Vis 3	0.269	0.281	0.313	0.291
	RH Vis 1	0.324	0.341	0.370	0.364
	RH Vis 2	0.298	0.345	0.363	0.350
Visual Network	RH Vis 3	0.342	0.346	0.395	0.367
	RH Vis 4	0.359	0.382	0.393	0.377
	RH Vis 5	0.368	0.378	0.389	0.373
	RH Vis 7	0.327	0.375	0.399	0.370
	LH Default Par 1	0.329	0.365	0.388	0.355
Default Mode	LH Default Par 2	0.298	0.325	0.359	0.326
Network	LH Default pCunPCC 1	0.287	0.312	0.349	0.327
	RH Default Par 2	0.346	0.356	0.373	0.366
	RH Default pCunPCC 2	0.231	0.271	0.307	0.315
	LH Limbic OFC 1	0.273	0.306	0.315	0.298
Limbic	LH Limbic TempPole 2	0.284	0.313	0.325	0.304
Network	RH Limbic TempPole 1	0.294	0.299	0.324	0.316
	RH Limbic OFC 1	0.286	0.297	0.317	0.306

LH, Left Hemisphere; RH, Right Hemisphere.

Table 8 indicates the node strength of the respective decreasing and increasing connections within different age groups. The ROIs which reflected changes with aging are from the Default Mode (DMN), Primary Somatosensory, and Prefrontal cortex (PFC) networks in the left hemisphere and the early Visual cortex and Ventral Attention Networks in the right hemisphere of the brain. Furthermore, age-dependent connections were from the Limbic Network in both the left and the right hemispheres.

Furthermore, by considering the node strength of the individual ROI from the Schaefer Atlas, it was possible to plot kernel density graphs to visualize the relationship between a brain region and age. These kernel density plots also show the direction of the age effect on the connectivity values. **Figure 5** illustrates the age associated functional connectivity using kernel density plots, within and between all pairs of networks. They show how the network is correlated (as depicted by the inverted parabolic line). In addition, the heat map in **Figure 5** shows the associations of age with functional connectivity within and between all pairs of networks.

From Figure 3, we can observe that the brain connectivity in the general population increase from the younger to the middle-aged group and shows a decrease in the old age group. We more specifically hypothesized that in adult and middle-aged persons from the general population, the brain ROIs show increase in functional connectivity.

It can be observed that the regions of the inverted-U functional connectivity network develop relatively slowly during the young age group this can be properly seen Ventral Attention Network in **Figure 5** but presents accelerated age-related degeneration at an old age in the kernel density plots. The inverted-U functional connectivity network can also be observed in other brain ROIs but the curve varies with the regions as we can see in the visual network, limbic network, default mode, somatosensory, and pre-frontal cortex network. **Figure 5** and Tables 7, 8 illustrate the age associated changes in the functional connectivity using kernel density plots, including intra- and inter-connectivity patterns for all pairs of networks. The kernel density plots show whether the brain network is positively or negatively associated with age (as depicted by the line). It can be observed from **Figures 5A**,**B**, that the functional connectivity of the pre-frontal cortex and somatosensory network is higher in the young age group compared to the old age group. On the contrary, (in **Figure 5D**) the visual network and (**Figure 5F**), limbic network sub-region intra-connected network showed a significant positive association with middle age and older age. With respect to network connectivity, we found both age-related increases and decreases in functional connectivity.

We can identify a decreasing trajectory (both rapid and linear) with aging in the PFC, primary somatosensory cortex, and DMN regions of the left hemisphere of the brain and an inverted U-shaped trajectory in the early Visual cortex and Ventral Attention regions of the right hemisphere of the brain.

## 4. Discussion

In this study, we proposed a deep learning framework based on the attention mechanism for the brain age prediction and age group classification leveraging static FC. The study reveals that attention-based CNN predicts the brain age more precisely when compared with the current state-of-the-art results (Li et al., 2018; Monti et al., 2020). The results reported shows that using convolutional neural architectures based on attention mechanisms yielded better performance for brain age estimation. We extended our study to a more meaningful 4-way classification (Young, Adult, Middle-Old, and Old age). We extracted aging-specific ROIs using integrated gradients and discussed the developmental and aging changes in the brain regions.

In this study, we have focused on using rsfMRI data to understand the functional changes with aging. The study from Cole et al. (2017) obtained age prediction accuracies with MAE as 4.16, *r*^2^ as 0.96, and RMSE as 5.31. (Jiang et al., 2020) exhibited the optimal age prediction accuracies with mean absolute errors (MAEs) of 5.55 years, 5.77 years, and 6.07 years from CNN based networks from FPN, DAN, and DMN regions. However, the work mainly focuses on structural MRI quantifying geometric structural properties such as the size and volume of a given structure or the thickness of a cortical area (e.g., gray matter). In the proposed study, we are interested in the functional connectivity across the whole brain providing information on how brain areas interact with one another. These changes may emerge as a consequence of structural changes with aging. We hypothesize that an integrated analysis of structural and functional neuroimaging data might help understand this relationship in future studies.

**Figure 4:**
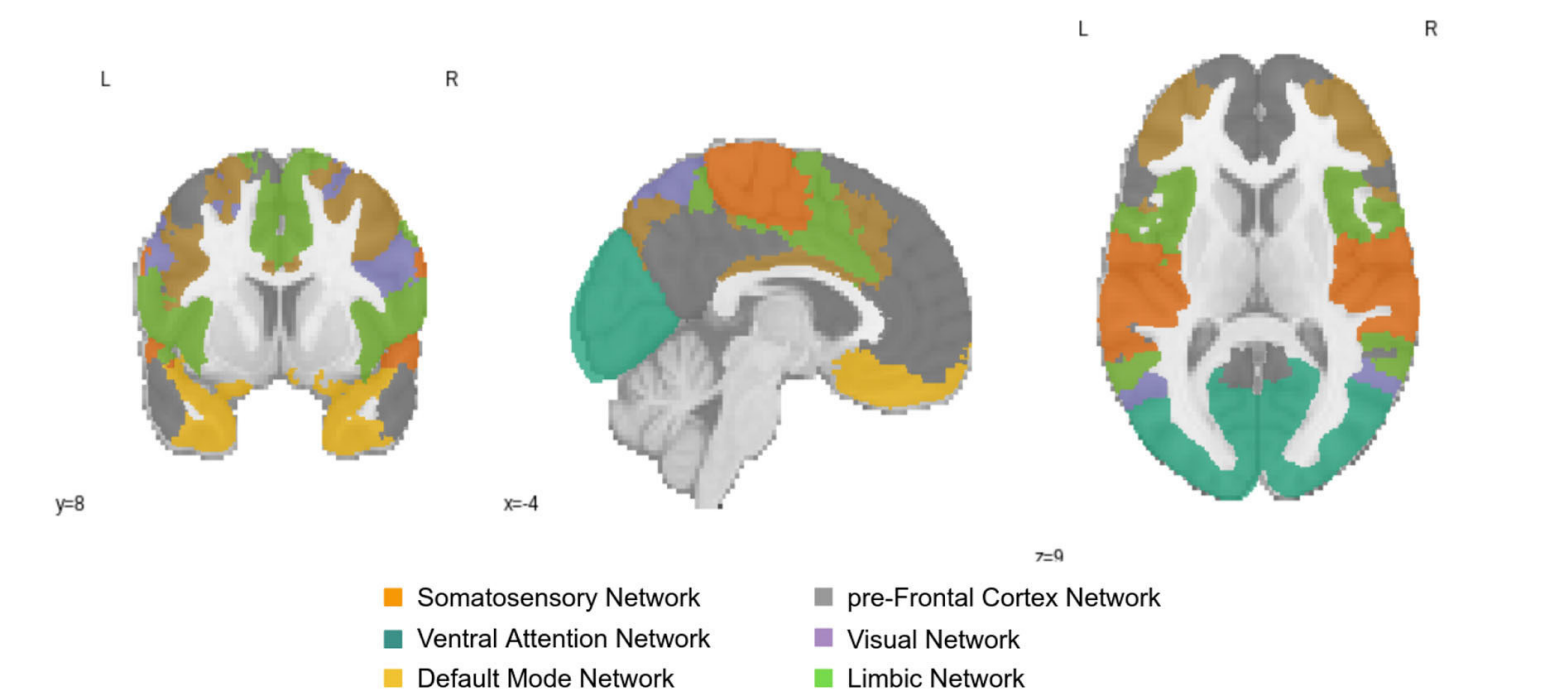
Resting-State Networks of Somatosensory Network; pre-Frontal Cortex Network; Ventral Attention Network; Visual Network; Default Mode Network; and Limbic Network; The color of the region represents the position of the region in the brain.

We explored whether the extracted ROIs from the integrated gradient were meaningful and how the correlation changes with aging. The ROIs which reflected changes with aging were from the Default Mode (DMN), Primary Somatosensory, and Prefrontal cortex (PFC) networks in the left hemisphere and the early Visual cortex and ventral Attention Networks in the right hemisphere of the brain. We found two relevant age-associated developmental trends: (i) a decreasing trajectory (both rapid and linear decrease) with aging in the PFC, primary Somatosensory and DMN regions of the left hemisphere of the brain, and (ii) an inverted U-shaped trajectory in the early Visual cortex and Ventral Attention regions of the right hemisphere of the brain. These ROIs are presented in Figure 4; which shows the resting-state region we studied.

The middle-aged and old group is associated with the increased magnitude of correlation values in Visual, Limbic, DMN, and Temporal Networks in between the network. Furthermore, older age was related to the decreased magnitude of correlation values between the Pre-frontal Cortex, Somatosensory network. The middle-aged group functional connectivity increased in Ventral Attention Network. We observed age-related increases and decreases in functional connectivity and positive and negative correlations between networks for between-network connectivity. Curiously, the old age group has reported increased functional connectivity between networks (Chan et al., 2014; Geerligs et al., 2015). It is theorized that these changes together within the brain networks reflect decreasing segregation. With aging, the brain changes its functional specialization (Chan et al., 2014; Geerligs et al., 2015). Our study adds to this by showing that this segregation is still subject to change in middle and old age.

These results arising from the analysis of the function are in line with the observation that these regions (Prefrontal Cortex; Somatosensory Network; Ventral Attention Network; Visual Network; Default Mode Network; Limbic Network) develop relatively late and slowly during adolescence and young adulthood, but show accelerated age-related degeneration in old age based on the brain structure analysis (Douaud et al., 2014). The DMN contributes to the mental exploration of social and emotional content and may contribute to adaptive behaviors, which tend to decrease as we age. Similarly, the PFC is an important site for working memory function and it also tends to reduce with age. Our results are consistent with the previous studies on aging (Chan et al., 2014).

**Figure 5:**
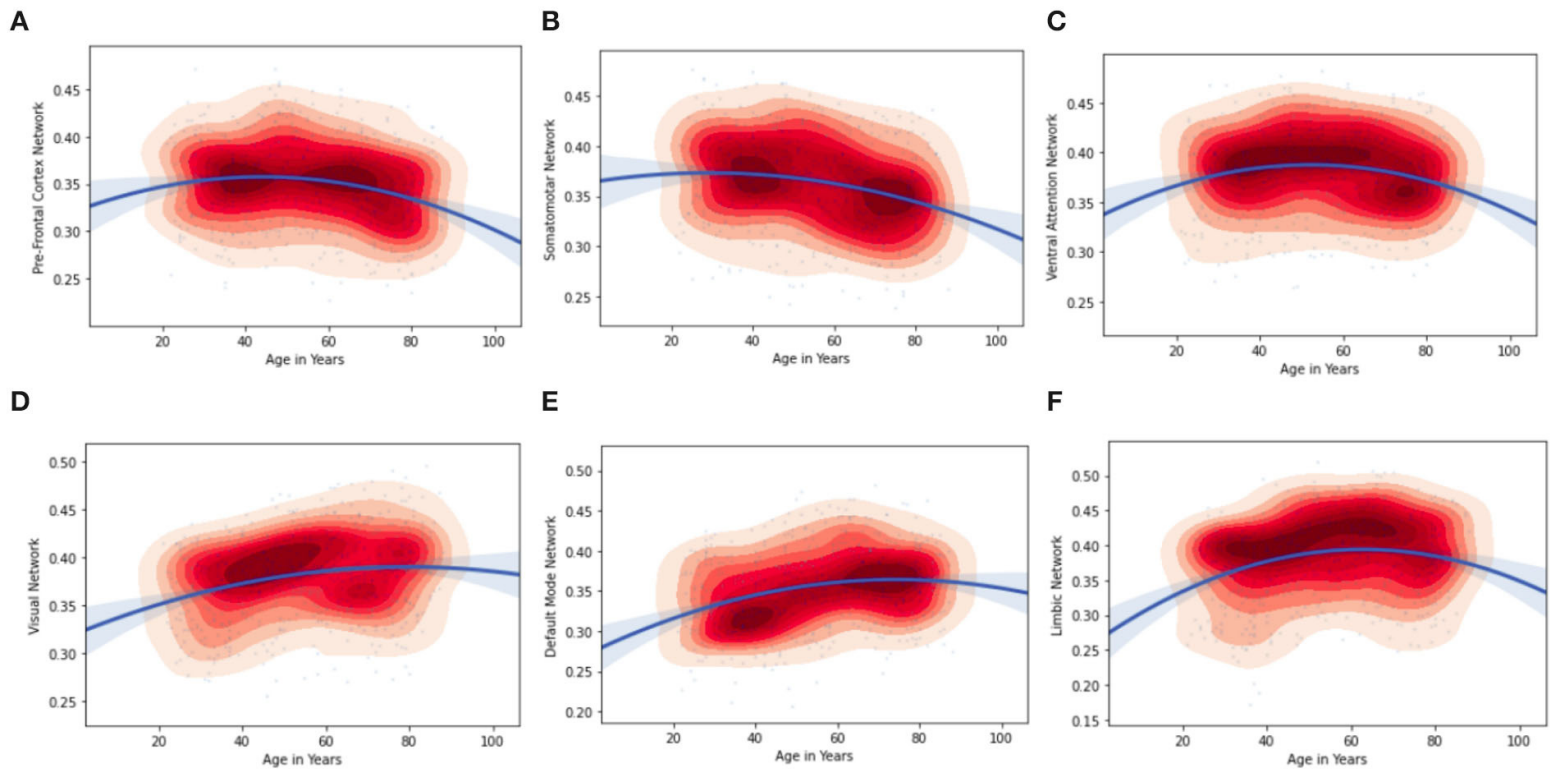
Age associations with correlation values of functional connectivity within networks. Kernel density plots visualize the distribution of the data (red = dense) and the direction of the age effect on the connectivity values; **(A)** pre-Frontal Cortex Network; **(B)** Somatosensory Network; **(C)** Ventral Attention Network; **(D)** Visual Network; **(E)** Default Mode Network; **(F)** Limbic Network.

In this study, we considered patterns of functional brain connectivity at the network level for individual ROIs in an aging population using resting-state fMRI. Our findings with older adults suggest that the strength of functional connectivity networks increases with older age affecting brain aging and suggesting that these extensive patterns of functional connectivity involving the default mode network, visual network, and limbic network, are relatively stable until the 70s or early 80s. Younger adults showed stronger connectivity within the prefrontal cortex, somatosensory network. The dorsal attention network and temporal network showed minimal changes across all the age groups which results in a weak age-association with functional connectivity on a network-level. The past studies that used the CamCan dataset to understand the changes in different regions of the brain with aging also suggest increased prefrontal cortex activity in the older age group in contrast to a decline being observed in their memory and other cognitive functions (Morcom and Henson, 2018), corroborating our results which are observed in the prefrontal cortex. This study reveals that this elevation is associated with non-specific neural responses instead of compensation i.e., it helps in maintaining cognitive function. On the other hand, increased activity in the prefrontal cortex might become less specific with aging. Another functional neuroimaging study suggests that there was reduced connectivity of the somatosensory network with aging (Wolpe et al., 2016). Our findings offer evidence in support of this statement (Figure 5 and Table 8). While the current study assumes that the participants were alert while resting with their eyes closed, there is a possibility of them falling asleep during the data acquisition period. This might influence the functional connectivity patterns. Future studies could take note of this possibility and address the issue appropriately.

Furthermore, our research study replicates previous demonstrations of an age difference in functional connectivity involving nodes of the DMN and shows that other networks are also important factors in brain aging (Meier et al., 2012; Li et al., 2018; Monti et al., 2020). Current results need to be seen in conjunction with the results of previous studies pointing out a decline in cognitive performance with age, possibly due to multiple incipient degenerative processes. Thus, functional connectivity may serve as a potential biomarker to study cognitive decline. However, neuropsychological tests are required in the future to corroborate the association between functional connectivity and cognitive decline.

In conclusion, the pipeline “Ayu” proposed in this study could be a basis for future studies focusing on functional connectivity and healthy aging, adding to our understanding of changes in the functional connectivity with respect to the aging brain in different age groups.

## Data availability statement

The original contributions presented in the study are included in the article/supplementary material, further inquiries can be directed to the corresponding authors.

## Author contributions

BR and PV: conceptualization and supervision. KB and AC: data analysis and investigation. KB, AC, BR, and PV: interpretation of results and writing–review editing. KB: writing–original draft. All authors have read and agreed to the published version of the manuscript.

## Conflict of interest

The authors declare that the research was conducted in the absence of any commercial or financial relationships that could be construed as a potential conflict of interest.

## Publisher's note

All claims expressed in this article are solely those of the authors and do not necessarily represent those of their affiliated organizations, or those of the publisher, the editors and the reviewers. Any product that may be evaluated in this article, or claim that may be made by its manufacturer, is not guaranteed or endorsed by the publisher.
